# Neuromodulation by soy diets or equol: Anti-depressive & anti-obesity-like influences, age- & hormone-dependent effects

**DOI:** 10.1186/1471-2202-12-28

**Published:** 2011-03-16

**Authors:** Crystal Blake, Kimberly M Fabick, Kenneth DR Setchell, Trent D Lund, Edwin D Lephart

**Affiliations:** 1 Department of Physiology and Developmental Biology and The Neuroscience Center, Brigham Young University, Provo, Utah 84602 USA; 2 Division of Pathology and Laboratory Medicine, Clinical Mass Spectrometry, Department of Pediatrics, Cincinnati Children's Hospital Medical Center, Cincinnati, Ohio 45229 USA; 3 Stoelting Co., 620 Wheat Lane, Wood Dale, Illinois, 60191 USA

## Abstract

**Background:**

Soy-derived isoflavones potentially protect against obesity and depression. In five different studies we examined the influence of soy-containing diets or equol injections on depression, serotonin levels, body weight gain (BW) and white adipose tissue (WAT) deposition in female Long-Evans rats at various stages of life [rats were intact, ovariectomized or experienced natural ovarian failure (NOF)].

**Results:**

In general, animals fed a soy-rich diet (Phyto-600) and/or administered equol (@ 5 mg/kg/day) displayed significant decreases in BW and WAT compared to a low-soy diet. When equol was injected alone (5 mg/kg/day), experiments 1, 4, and 5 demonstrated that body weight was significantly decreased. Equol has body weight control effects in females that are dependent on ovarian status and/or age of diet initiation. Experiments 1-4 all displayed no significant differences in depressive-related behavior as measured by the Prosolt forced swim test (PFST) when soy-rich (Phyto-600) or low-soy diets (Phyto-low) or equol treatments (5 mg/kg/day) were tested in female rats at various ages or hormonal status. Results of all the experiments are not presented here due to space limitations, but data from experiment 5 are presented. From conception female rats were exposed to either: a) a soy-rich (Phyto-600) or b) low-soy diet (Phyto-low). After 290 days all rats experienced NOF. At 330 days-old the animals were examined in the Porsolt forced swim test (PFST). One month later a second PFST was performed [after Phyto-low fed animals were injected with equol (5 mg/kg/day) for one week prior to the second PFST]. At the first PFST, serotonin and mobility levels were significantly decreased in the Phyto-low fed animals compared to animals that consumed the Phyto-600 diet. After equol injections at the second PFST, mobility and serotonin levels significantly increased in aged NOF rats fed the Phyto-low diet (to levels comparable to Phyto-600 fed animals).

**Conclusions:**

Consumption of dietary isoflavones or equol exposure in rats has body weight controlling effects and equol specifically may have antidepressant potential dependent upon diet initiation and/or dosage of treatments. The current study demonstrates that equol is able to decrease body weight, abdominal WAT, and depressive-related behavior. While other factors and mechanisms may play a role, in part, the present results provide a greater understanding of how isoflavonoid molecules modulate the brain's influence on behavior.

## Background

Following the 2002 Women's Health Initiative (WHI), concerns about hormone replacement therapy (HRT) amplified interest in alternative therapies for the treatment of menopausal symptoms [1-3]. Polyphenols are plant-derived substances that are available as dietary supplements [4,5]. The best-studied are the phytoestrogens, which include isoflavones, lignans, and coumestans [5-7]. Of these, isoflavones are the type most commonly found in both rodent and human diets [8] and have shown potential as treatments for a number of age-related disorders, such as, cardiovascular disease, breast and prostate cancers [9].

Isoflavones, such as daidzein and genistein, are selective estrogen receptor modulators (SERM) or biochemical compounds that are able to agonize or antagonize estrogen receptors (ER) [10]. Daidzein and genistein are able to bind both ERα and ERβ but demonstrate a higher affinity for ERβ [8,10]. Moreover, daidzein's intestinally produced metabolite, equol, has a higher affinity for ERβ than daidzein or genistein [11]. This chemical characteristic of equol to bind estrogen receptor subtypes indicates potential as an alternative treatment to HRT for menopausal symptoms.

Menopausal symptoms such as hot flashes, insomnia, depression, and weight gain are associated with the loss of ovarian steroid production and availability [1,12-14]. HRT effectively decreases these symptoms and is the only current treatment approved by the FDA [1]. However, since the 2002 WHI raised the concern that HRT increases cancer risk, researchers, health professionals, and postmenopausal women are looking for other ways to treat menopausal symptoms [1,3]. Due to the varying actions and distributions of ERα and ERβ, specific treatments can now be administered that target either receptor [15,16]. For example, isoflavonoids via ERβ, due to this estrogen subtype's distribution and interactions with other molecules and receptors, potentially may play an important role as an HRT alternative [7,15,16].

In a series of reports we demonstrated that soy-derived isoflavones can significantly decreased body weight, adipose tissue deposition, skin tail temperature and produce anxiolytic effects [17-20]. Notably, in a comprehensive study the beneficial influences of soy-derived isoflavones via diet were examined that included body weight, adipose tissue deposition, food and water intake, metabolic hormones (i.e. leptin, insulin, thyroid (T3) and glucose levels), brain neuropeptide Y (NPY) levels in hypothalamic regions, heat production [in brown adipose tissue (BAT) quantifying uncoupling protein (UCP-1) mRNA levels] and core body temperature [19].

Thus, this stimulated us to examine whether isoflavones (particularly equol) may be used as both an antidepressant and an anti-obesity treatment in intact female rats and two animal models of menopause (ovariectomy and natural ovarian failure).

## Methods

### General methodology

In five different experiments we examined the influence of soy-containing diets and equol administered via subcutaneous (s.c.) injections on depressive-like behaviors, serum serotonin levels, weight gain (BW) and white adipose tissue (WAT) deposition in female Long-Evans rats at various stages of postnatal life.

### Animals, mating and housing

All animals had free access to food (diet treatment) and tap water. Only the animals in Experiment 3 were not bred at our facilities. [The Experiment 3 rats were purchased ovariectomized (at approximately 45-50 days-old) from Charles River Laboratories (CRL) and following surgery/recovery the animals were shipped to our facilities.] All other male and female rats purchased from the supplier (CRL, at approximately 50-55 days-old) were allowed time to adapt to their new surroundings and diet treatments before being bred in our animal facilities at approximately 90 days old for each experiment (outlined below). Mating was performed by a protocol previous reported by our laboratory [18]. In brief, all of the males were fertile and each male inseminated 2 to 4 females by diet treatment by experiment. After birth the pups remained on the same diets as their mothers. At age 21 days the rats were weaned and housed in cages [standard shoebox (20 cm high × 24 cm wide × 40 cm deep) containing wood/shaving-chip bedding] with 3 to 4 animals per cage by treatment. At 40 days of age the animals were separated (2 animals per cage by treatment) until 60 days of age when 1 animal per cage defined the housing protocol until the end of all the experiments.

### Diets

From conception (except for Experiment 3 where animals were ovariectomized at that supplier, see above; i.e., the diet for these animals from conception until arrival at our facilities was a Phyto-200 diet, see results section) the female rats were exposed to either a soy-rich (Phyto-600) diet that contained 600 ppm of isoflavones (Harlan Teklad 8604, Madison, WI, USA) or a low-soy (Phyto-low) diet that contained approximately 10 ppm of isoflavones (Zeigler Bros. Inc., Gardners, PA, USA), (catalog # 541200-12-00, Rodent PHY RDC). Each diet protocol is outlined below by experiment number and descriptor. Furthermore, the diet formulations (ingredient list) have been published previously [17-19]. Notably, for the soy-rich (Phyto-600) diet, soy meal is the first ingredient and this diet treatment does not contain a soy protein isolate. This was accomplished by placing the males and females that were bred at our animal facilities on the respective diets by each experimental design (as outlined above).

### Injections

In general, before running the last Porsolt Forced Swim Tests rats received subcutaneous (s.c.) injections (@ nape of the neck) of either dimethyl sulfoxide @ 100% (DMSO, HPLC grade; Sigma Chemical Co., St. Louis, MO, USA) that served as controls or 5.0 mg/kg body weight of equol (Central Glass Co., Kuensebeck, Germany) in DMSO. Daily injection volume was 0.1 cc (see specific experiment number below for injection schedule). While the length of time injected differed between experiments the equol concentration remained at 5 mg/kg in each experiment. [Notably: in other experiments an equol dose of 2.5 mg/kg yielded similar results].

### Ovariectomy

Animals in Experiment 4 were ovariectomized in our laboratory between 96-100 days of age. Animals were anesthetized with a combination of (3 cc buprenex, 2 cc domitor and 5 cc of ketamine) at 0.05 cc per 100 grams body weight. Body temperature was maintained with a warm water blanket. Both ovaries were surgically removed (with the assistance and supervision of our university veterinarian), the incision was closed with sterile surgical staples and animals were given acetaminophen [20 ml of pediatric elixir (32 mg/ml) per 500 ml tap water] in their drinking water for approximately 4-5 days post-surgery during recovery.

### Porsolt forced swim test

We utilized the Porsolt forced swim test (PFST) that has become a standard antidepressant test in pharmaceutical laboratories worldwide and in academic laboratories for investigating the neurobiology of depression and of antidepressant agent action [21,22]. It tests depressive-like behaviors by quantifying an animal's mobility and immobility and associated behaviors.

Each rat was placed in a plastic cylindrical container (diameter, 19 cm, height, 43 cm) containing water at a depth of 35.6 cm for 8 minutes. Rats were unable to touch the bottom of the container with their tails [23]. The behavior was then recorded both by a video camera above and experienced observers to the side. Swimming speed, distance, time spent both mobile and immobile were recorded. Animals that express fewer depressive-related behaviors spend more time mobile, swim farther and have a higher overall average swimming speed. Animals that express depressive-related behaviors, spend more time immobile, swim for a shorter distance, and have an overall slower average swim speed. All behaviors were analyzed by Anymaze^® ^computer software (Stoelting Co., Wood Dale, IL, USA).

### Body weight, white adipose tissue (WAT) deposition and blood collection

Body weight (in grams) was recorded at different intervals for each experiment (see below). At the end of each experiment, trunk blood was collected, serum prepared and then stored at -20°C until assayed for serotonin or isoflavone concentrations. WAT was dissected from just below the diaphragm to the base of the pelvic cavity and recorded in grams.

### Serotonin ELISA

Serum serotonin levels were quantified using ELISA kits (Cat# IB89527-121610) purchased from Immuno Biological Laboratories (Minneapolis, MN, USA). Serum was analyzed for all of the experiments except experiment 3.

### Serum phytoestrogen levels

Serum was analyzed via gas chromatography/mass spectrometry (GC/MS) and the circulating isoflavones: genistein, daidzein, and equol concentrations were determined (ng/ml) using internal control standards, as previously reported in other studies by our laboratory [18,19].

### IACUC approval

All methods associated with animal use/surgery were approved by the Institutional Animal Care and Use Committee at Brigham Young University.

#### Treatments

##### Experiment 1: Phyto-low diet exposure from conception to 120 days post-birth with diet change of one-half of the animals to a Phyto-600 diet until 210 days of age

**Figure 1:**
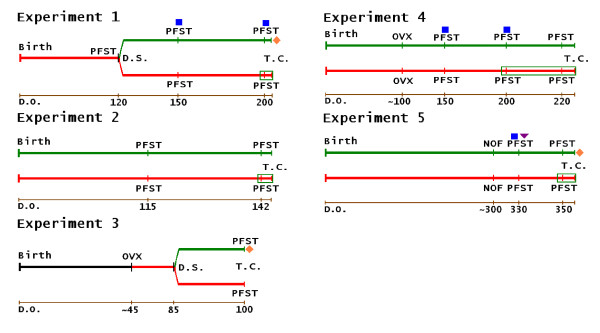
**Experiments Summary Figure**. D.S. = diet-switched; PFST = Porsolt forced swim test; T.C. = tissue collection; OVX = ovariectomy; NOF = natural ovarian failure; Red bar = Phyto-low diet; Green bar = Phyto-600 diet; Black bar = supplier diet; Green box = equol injections; orange diamond = white adipose tissue significantly less; blue square = body weight significantly less; down purple triangle = time immobile significantly lower, D.O. = days old; ~ = approximately.

This experiment examined the influence of diet on body weight, WAT, serotonin levels, isoflavone levels, and behavior as measured in the PFST. From conception to young adulthood, 23 female rats representing 6 litters were fed the Phyto-low diet. At age 120 days animals were run on the Porsolt forced swim test (PFST) to obtain baseline levels for depressive-like behaviors in Phyto-low fed female rats. Following this PFST, 12 animals were switched to a Phyto-600 diet while 11 animals remained on the original Phyto-low diet. The animals remained on these diet treatments (Phyto-600 or Phyto-low) until the end of this experiment. When animals were age 155 days they were run a second time on the PFST and behavioral parameters were recorded. From age 194 to 200 days, the Phyto-low fed animals received 5 mg/kg (body weight) injections of equol while the Phyto-600 animals received vehicle-DMSO injections. At age 200 days the animals were run on a third PFST and behavioral parameters were recorded. The dietary and treatment injections continued until the animals were sacrificed at age 210 days when body and WAT weights were recorded and blood was collected for serotonin and isoflavone quantification (Figure 1).

##### Experiment 2: Phyto-low or Phyto-600 diet exposure by treatment group from conception to 145 days of age

This experiment examined the influence of a low soy-containing diet (Phyto-low) or a soy-rich (Phyto-600) diet from conception to adulthood (145 days-old) in intact rats for the study parameters. Animals were fed either the Phyto-low (n = 8, representing 4 litters) or the Phyto-600 (n = 7, representing 3 litters) diet. Animals were weighed three times a week. When the rats were 115 days-old the rats were run on the first PFST. After 3 weeks the Phyto-low fed animals were injected for 4 days (136 to 142 days-old) prior to second PFST with 5 mg/kg of equol while the Phyto-600 fed animals received vehicle-DMSO injections. At 142 days-old the animals were run on a second PFST. These injections continued until the animals were sacrificed at 145 days-old when body and WAT weights were recorded and trunk blood was collected for quantification of serotonin and isoflavones concentrations (Figure 1). [Note: the animals in experiment 2 were culled (due to the large number of litters) which excluded the lightest and heaviest animals by diet treatment before 145 days of age].

##### Experiment 3: Phyto-200 (200 ppm of isoflavones) diet exposure from conception to 45 days post-birth at the supplier (ovariectomized). Upon arrival (45 days of age) all animals were changed to the Phyto-low, then one-half of the animals where changed to a Phyto-600 diet at 85 days of age until 100 days old

This experiment examined initially the influence of a low soy-containing diet (Phyto-low) diet from approximately 50-55 days of age until 80-85 days of age in twenty-two ovariectomized rats purchased from Charles River Laboratories. The animals were weighed daily during this experiment. At approximately age 85 days eleven animals were changed to a soy-rich (Phyto-600) diet while the other eleven animals remained on the original soy-low or Phyto-low diet for approximately two weeks. When the animals were approximately age 100 days they were tested in the PFST. The animals were then sacrificed and body and WAT weights were recorded and blood collected for subsequent analysis of isoflavone concentrations (Figure 1).

##### Experiment 4: Phyto-low or Phyto-600 diet exposure from conception to approximately 100 day old when all animals were ovariectomized. Then the animals remained on their respective diet treatments (Phyto-low or Phyto-600) until 222 days of age

This experiment examined the influence of either a low soy-containing diet (Phyto-low) or a soy-rich (Phyto-600) diet from conception to adulthood (222 days-old) on the study parameters. Animals were initially intact rats and later ovariectomized (in our laboratory) as young adults. The females were fed either the Phyto-low (n = 8 from 4 litters) or Phyto-600 (n = 8 from 3 litters) diet. Animals were weighed three times a week. Animals were ovariectomized at approximately age 100 days. At age 150 days animals were run on the PFST and behavioral parameters were recorded. The animals continued on their respective diet treatments until age 200 days when they were run on a second PFST. However, four days prior to this second PFST (from age 197 to age 200 days) the Phyto-low fed animals received s.c. injections of 5 mg/kg of equol and the Phyto-600 fed animals were administered vehicle-DMSO injections. After the second PFST animals continued to received daily s.c. injections by dietary treatments until being run on a third PFST age 220 days. The injections by dietary treatments continued until animals were sacrificed at age 222 days when the study parameters were collected (body/WAT weights) and quantified (serum serotonin and isoflavone levels) (Figure 1).

##### Experiment 5: Phyto-low or Phyto-600 diet exposure from conception to until the end of the experiment at 365 days of age. All females experienced NOF at around 295 days old

This experiment examined the influence of a low soy-containing diet (Phyto-low; n = 6) or a soy-rich (Phyto-600; n = 8) diet from conception to mid-age on the study parameters in intact rats (representing at least 2 to 3 litters by dietary treatment) that experienced natural ovarian failure (NOF). Intact, Long-Evans female rats were exposed to a Phyto-low diet or a Phyto-600 lifelong from conception to mid-age (> 300 days-old). The animals were weighed at selected time intervals in this experiment. At approximately 300 days-old all animals (regardless of diet treatment) experienced NOF as detected by vaginal histological/microscopic examination of the epithelial cells over a 10-day interval (from 295 to 305 days-old). Twenty-seven days later approximately one half of the animals in each treatment group had blood collected via tail vein for baseline serotonin and isoflavone levels. At 330 days-old the animals (by dietary treatment group) were run on the first PFST. Twenty-four days later (@ approximately 353 days-old) the Phyto-low fed animals received daily s.c. injections of equol (@ 5 mg/kg) while the Phyto-600 fed animals received vehicle-DMSO injections for 7 consecutive days. Two hours after the last injection on the seventh day the animals were again run for a second time on the PFST (by diet and injection treatments). Injections continued until animals were sacrificed at 365 days-old; body and WAT weights were recorded and trunk blood was collected for quantification of serotonin and isoflavones concentrations (Figure 1).

### Statistical analysis

Two-sample student t-tests with repeated measures were used to compare each of the parameters examined between the two treatment groups in each experiment or non-parametric analysis with repeated measures, where appropriate. All statistics were run using the Minitab statistical software, p < 0.05 was considered significant. All results are presented as MEANS ± SEM in all of the graphs and significant differences are marked.

## Results

Results from the overall study are displayed in Figure 1 with each experiment's major points of significance and corresponding age highlighted.

### Experiments 1, 2, 3 and 4

#### Body weight and WAT: Body weight changes and white adipose tissue deposition levels over each treatment period for experiments 1, 2, 3 and 4 are displayed in Figures 2, 3, 4, 5

**Figure 2:**
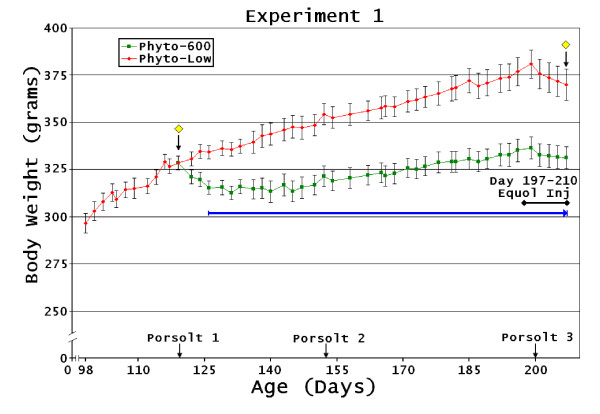
**Intact Diet-Changed Female Body Weight Changes**. Experiment 1: (Phyto-low diet exposure from conception to 120 days post-birth with diet change of one-half of the animals to a Phyto-600 diet until 210 days of age). Yellow diamond indicates diet treatment start and finish dates; blue line indicates time body weights significantly different between treatment groups.

**Figure 3:**
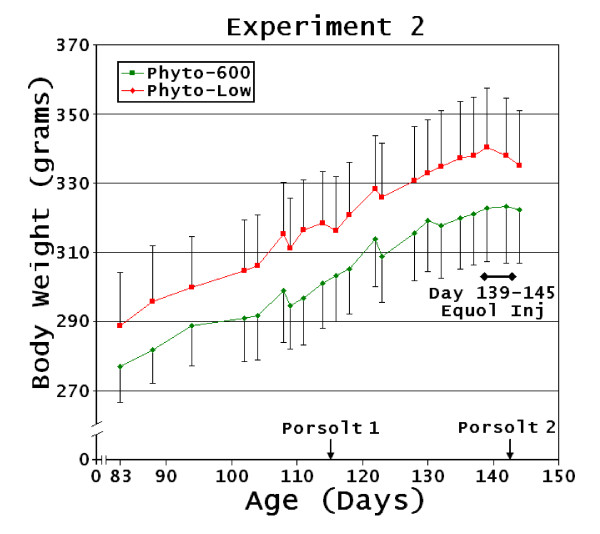
**Intact Lifelong Diet Female Body Weight Changes**. Experiment 2: (Phyto-low or Phyto-600 diet exposure by treatment group from conception to 145 days of age). No significant differences were observed in body weights between the treatments but values tended to be lower in the Phyto-600 vs. Phyto-low animals and body weights in the Phyto-low fed animals declined with equol injections (black line with end diamonds).

**Figure 4:**
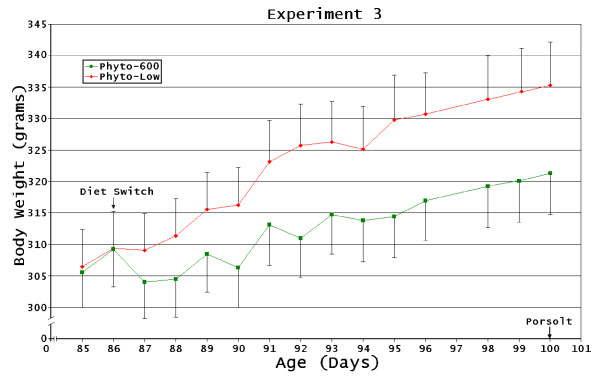
**Ovariectomized Diet-Changed Female Body Weight Changes**. Experiment 3: [Phyto-200 diet exposure from conception to 45 days post-birth at the supplier (ovariectomized). Upon arrival (45 days of age) all animals were changed to the Phyto-low, then one-half of the animals where changed to a Phyto-600 diet at 85 days of age until 100 days old]. No significant differences were observed in body weights between the treatments but values tended to be lower in the Phyto-600 vs. Phyto-low animals.

**Figure 5:**
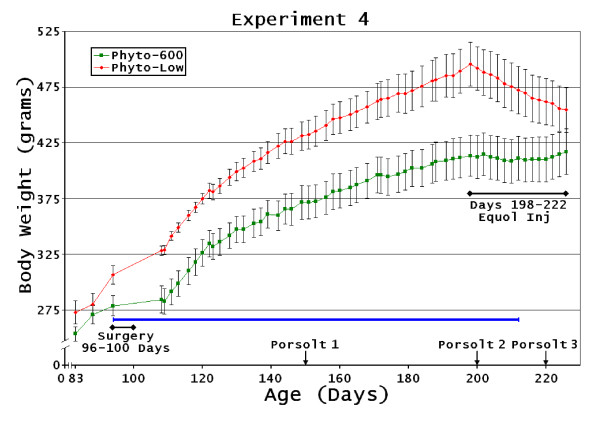
**Ovariectomized Lifelong Diet Female Body Weight Changes**. Experiment 4: Blue line indicates time body weights significantly different between treatment groups. (Phyto-low or Phyto-600 diet exposure from conception to approximately 100 day old when all animals were ovariectomized. Then the animals remained on their respective diet treatments (Phyto-low or Phyto-600) until 222 days of age). Equol injections (black line with end diamonds)

In each experiment, prior to each PFST or tissue collection, body weights were analyzed and the corresponding values (for BW, WAT, and WAT/BW) and significance are shown in Table 1.

**Table 1:** Body Weight Prior to Each Porsolt Test and Tissue Collection Body Weight, WAT and WAT/BW (grams, MEAN ± SEM)

Experiment	Diet	1^st ^PFST	2^nd ^PFST	3^rd ^PFST	T.C. BW	T.C. WAT	WAT/BW
1	Phyto-low	330.4 ± 5.9	354.0 ± 5.7**	375.6 ± 7.3**	369.9 ± 80.4**	20.1 ± 1.7*	0.054 ± 0.004*
	Phyto-600	327.6 ± 5.1	326.4 ± 5.4	332.6 ± 6.7	331.3 ± 6.4	13.1 ± 1.2	0.039 ± 0.003

2	Phyto-low	318.5 ± 15.0	**338.2 ± 16.5**	N/A	335.1 ± 15.8	16.1 ± 2.4	0.047 ± 0.005
	Phyto-600	301.1 ± 12.9	323.2 ± 16.3		322.3 ± 15.2	11.9 ± 2.5	0.036 ± 0.006

3	Phyto-low	336.0 ± 7.3	N/A	N/A	336.0 ± 7.3	16.0 ± 0.9	0.042 ± 0.005
	Phyto-600	319.1 ± 6.8	N/A	N/A	319.1 ± 6.8	11.4 ± 0.7#	0.032 ± 0.004

4	Phyto-low	431.0 ± 12.6*	487.1 ± 20.6*	**454.4 ± 56.6**	453.5 ± 19.3	23.4 ± 5.1	0.049 ± 0.009
	Phyto-600	371.5 ± 15.4	414.6 ± 12.3	417.0 ± 57.8	413.6 ± 20.2	20.2 ± 2.0	0.048 ± 0.010

5	Phyto-low	526.5 ± 25.9*	**507.9 ± 21.2**	N/A	505.6 ± 21.0	32.1 ± 3.9#	0.063 ± 0.006#
	Phyto-600	469.1 ± 21.3	472.1 ± 19.6	N/A	471.7 ± 19.0	23.3 ± 2.7	0.049 ± 0.005

Comparing diet and/or equol treatments within a column category: ** p < 0.001; * p < 0.01; # p < 0.05; PFST = Porsolt forced swim test, N/A = not assayed, T.C. = tissue collection; BW = body weight; WAT = white adipose tissue deposition, **Bold values **= time of equol injection.

In general, the animals fed the Phyto-600 diet displayed lower body weight gain. However, statistically significant differences were only seen following the diet change in experiment 1 and following ovariectomy in experiment 4.

In experiment 1: Phyto-low diet exposure from conception to 120 days post-birth with diet change of one-half of the animals to a Phyto-600 diet until 210 days of age.

The Phyto-600 animals displayed significantly decreased weight gain following the change to the Phyto-600 diet (Figure 2). Within 6 days these animals weighed significantly less than the Phyto-low animals (p < 0.031) which continued to the end of the experiment. WAT values were also significantly lower in the Phyto-600 animals (p < 0.003).

In experiment 4: Phyto-low or Phyto-600 diet exposure from conception to approximately 100 day old when all animals were ovariectomized. Then the animals remained on their respective diet treatments (Phyto-low or Phyto-600) until 222 days of age.

The Phyto-600 females had significantly lower body weights following ovariectomy (p < 0.01; Figure 5). This difference continued until animals were 212 days-old at which point these there was no longer any significant difference between treatment groups (for body or WAT weights) due to the equol injections in the Phyto-low group near the end of the experiment.

The Phyto-600 animals in experiment 2 (Phyto-low or Phyto-600 diet exposure by treatment group from conception to 145 days of age) and in experiment 3 [Phyto-200 diet exposure from conception to 45 days post-birth at the supplier (ovariectomized). Upon arrival (45 days of age) all animals were changed to the Phyto-low, then one-half of the animals where changed to a Phyto-600 diet at 85 days of age until 100 days old] had lower average body weights than the Phyto-low fed animals but these differences were not significant. The animals in experiment 2 were culled (due to the relatively large numbers of animals initially) to remove any outliers prior to treatment and had the shortest treatment periods (see methods). We believe that had treatment continued the Phyto-600 animals would have displayed significantly lower body weights due to the observed change in slopes of the body weights.

In experiment 3 Phyto-600 WAT was significantly lower (p < 0.001); however when normalized to body weight (WAT/BW) this was not significant (p < 0.086). Equol injections in the Phyto-low animals decreased body weight as shown in Figure 2, 3, and 5. However, only in experiment 4 [Phyto-low or Phyto-600 diet exposure from conception to approximately 100 day old when all animals were ovariectomized. Then the animals remained on their respective diet treatments (Phyto-low or Phyto-600) until 222 days of age)] were equol injections administered long enough (approximately two weeks) to significantly demonstrate this weight-reducing influence.

##### Porsolt forced swim test

Experiments 1-4 demonstrated no significant differences between treatment groups for any behavioral study parameters in any of the PFSTs performed in these experiments (Figure 1).

##### Serum isoflavone levels

In experiments 1-3 serum levels of daidzein, genistein, and equol were all significantly lower in the Phyto-low animals (p < 0.001-0.008). Serum equol levels of the equol-injected Phyto-low animals were less than half the levels of the intestinally produced equol in the Phyto-600 fed animals (Table 2). In experiment 4, genistein and daidzein levels were significantly less in the Phyto-low females (p < 0.005 and p < 0.006 respectively) while equol levels were significantly greater (p < 0.002). In each of these experiments equol was the major circulating phytoestrogen in both treatment groups while genistein and daidzein represented lower percentages of the total phytoestrogen content.

**Table 2:** Serum Levels of Isoflavones, Genistein and Daidzein, and Isoflavonoid Equol (all values in ng/ml ± 1.0)

Experiment	Diet	Genistein	Daidzein	Equol	Total (G+D+E)
1	Phyto-low	8.1 ± 0.6*	4.8 ± 0.7*	439.1 ± 80.5*	452.0 ± 80.4*
	Phyto-600	45.3 ± 8.4	34.9 ± 6.7	932.3 ± 121.4	1012.5 ± 135.5

2	Phyto-low	7.1 ± 1.4*	7.0 ± 0.8*	345.5 ± 97.1*	359.5 ± 96.5*
	Phyto-600	32.1 ± 11.8	30.5 ± 5.1	753.5 ± 76.6	816.1 ± 81.0

3	Phyto-low	5.3 ± 1.1*	3.1 ± 0.3*	4.1 ± 0.4*	12.4 ± 1.4*
	Phyto-600	48.5 ± 8.6	38.2 ± 7.0	495.6 ± 33.3	582.3 ± 31.2

4	Phyto-low	8.3 ± 0.4*	8.0 ± 1.7*	779.2 ± 60.4 #	792.2 ± 63.7 #
	Phyto-600	36.0 ± 7.8	40.4 ± 9.2	415.1 ± 64.9	491.5 ± 76.3

5	Phyto-low				
	**Before**	4.6 ± 0.8*	3.1 ± 0.3*	4.0 ± 0.4*	11.1 ± 1.2*
	**After**	3.7 ± 0.5*	3.1 ± 0.3*	376.3 ± 92.9	383.3 ± 92.0
	Phyto-600				
	**Before**	31.4 ± 6.3	36.1 ± 7.1	339.7 ± 13.7	407.3 ± 27.0
	**After**	25.2 ± 5.0	34.2 ± 6.5	321.6 ± 10.0	381.0 ± 21.0

* Phyto-low value significantly lower than Phyto-600 value; # Phyto-low value significantly higher than Phyto-600

value; ** Phyto-low Before value significantly lower than After value; **Before **refers to serum measured prior to 1^st^

Porsolt test; **After **refers to serum measured after 2^nd ^Porsolt test.

##### Serum serotonin levels

There were no significant differences in serum serotonin values between treatment groups for experiments 1-4 (Phyto-low values ranged between 262.9 + 107.3 to 544.9 + 205.9 vs Phyto-600 values ranged between 317.2 + 129.6 to 444.5 + 168.0). These values were obtained at the end of each experiment; prior serotonin values were not determined (data not shown graphically).

#### Experiment 5: Phyto-low or Phyto-600 diet exposure from conception to until the end of the experiment at 365 days of age. All females experienced NOF at around 295 days old

##### Body weight and WAT

Animal body weight measurements were only recorded at specific points during this experiment and these points and respective ages are marked in Figure 1. At the first PFST, which occurred approximately a month after NOF, the Phyto-600 fed animals had significantly lower body weights than the Phyto-low animals (p < 0.05; Table 1). One week prior to the second PFST, the Phyto-low animals received daily equol injections. At the second PFST, animal body weights were no longer significantly different between treatment groups. However, WAT deposition was still significantly greater in the Phyto-low fed animals (p < 0.05) and this trend continued even when WAT was normalized to body weight WAT/BW (p < 0.05; Table 1).

##### Porsolt forced swim test

**Figure 6:**
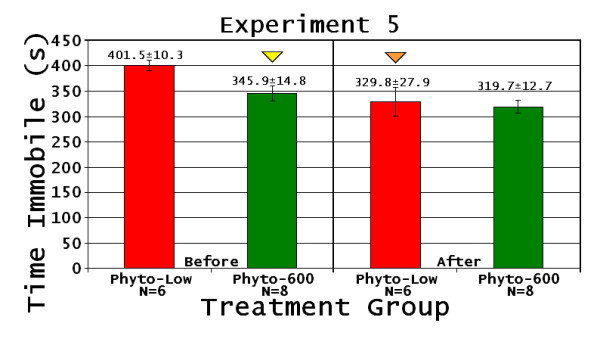
**Natural Ovarian Failure Female Time Immobile**. Experiment 5: (Phyto-low or Phyto-600 diet exposure from conception to until the end of the experiment at 365 days of age. All females experienced NOF at around 295 days old).Down yellow triangle Phyto-600 time immobile significantly lower than Phyto-low animals. Down orange triangle Phyto-low After time immobile significantly decreased compared to Before time immobile. Before refers to 1^st ^Porsolt test, After refers to 2^nd ^Porsolt test, all data expressed as the mean ± the standard error of the mean (SEM).

In the first PFST, time immobile (Figure 6), swimming distance (data not shown), and overall average speed (data not shown) were significantly higher in the Phyto-600 fed animals compared to the Phyto-low fed animals (p < 0.005). This is the only experiment where a dramatic and significant difference between lifelong exposure to the diet treatments occurred. Equol injection was also the most effective in this experiment. For example, following the equol injections, immobility significantly decreased in the Phyto-low fed animals compared to pre-equol injection values (p < 0.05; Figure 6).

##### Serum isoflavone levels

All isoflavone values are shown in Table 2. Serum levels of daidzein, genistein, and equol (daidzein's intestinal metabolite) were all significantly lower in the Phyto-low animals compared to the Phyto-600 animals prior to the first PFST (p < 0.001). Following the second PFST only serum levels of daidzein and genistein were significantly lower in the Phyto-low animals compared to the Phyto-600 animals (p < 0.001). Serum equol values were not significantly different between treatment groups following the second PFST. Equol represented the highest percentage of the total isoflavone concentrations.

##### Serum serotonin levels

**Figure 7:**
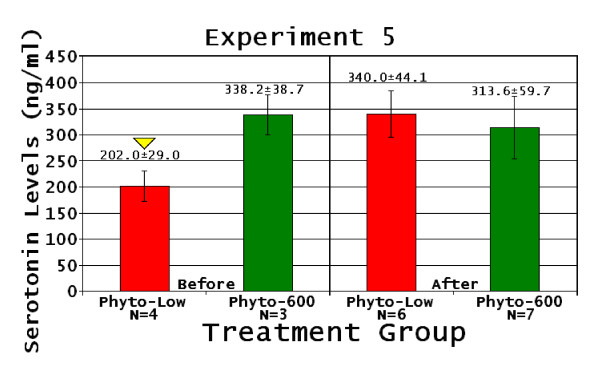
**Natural Ovarian Failure Female Serum Serotonin Levels**. Experiment 5: Down yellow triangle serotonin Before levels significantly lower than After levels. Before refers to serum drawn prior to 1^st ^Porsolt test, After refers to serum analyzed after 2^nd ^Porsolt test, all values expressed in ng/ml ± 1.0.

The Phyto-low fed animals displayed lower serum serotonin values prior to the first PFST vs. Phyto-600 values (Figure 7). However, statistical significance could not be numerically obtained between treatment groups due to the low number of animals whose blood was drawn prior to equol injections (n = 3 Phyto-low and n = 4 Phyto-600). We believe these findings are noteworthy because the Phyto-600 values were over 100 ng/ml greater (or 1.7 times higher) than the Phyto-low values. Furthermore, following equol injections, the Phyto-low serotonin values significantly increased compared to pre-injection levels (p < 0.042) and were comparable to Phyto-600 values (Figure 7).

## Discussion

In general, this study demonstrates that either dietary isoflavone exposure or equol alone effectively decreased body weight and WAT deposition in females regardless of ovarian status, which is similar to that reported previously [17-20,24]. Of note, the primary isoflavone present in the serum of rats fed a soy-rich diet is equol (Table 2) while genistein and daidzein represent smaller percentages of the total isoflavone count [18,19]. When equol is injected alone, experiments 1, 4, and 5 demonstrate that body weight still decreased. Equol has body weight controlling effects in female rats is dependent on age and initiation of the diet treatments. Neurobehavioral influences may underlie the present effects due to ovarian status and the dosage of isoflavonoids administered [25]. However, while equol's mechanism of action is unknown, evidence to support the role of ERβ's anti-adiposity in animals and humans has been reported [26,27]. Moreover, animals fed soy-containing diets or administered genistein appears to alter food intake and metabolic hormones, body weight and adipose tissue deposition as well as lipid uptake into adipocytes by decreasing lipoprotein lipase (LPL) activity [18,19,28-32]. Daidzein also appears to decrease LPL activity, increase lipolysis, and inhibit adipogenesis via estrogenic pathways [28,33,34]. Furthermore, unpublished data by our lab demonstrates that equol's actions can be blocked by an ER antagonist. Tamoxifen, a SERM able to antagonize ERs [35] is able to block the positive influence of equol in human monolayer fibroblasts.

This study also demonstrated that equol decreases depressive-related behavior in NOF females. Prior to equol injections in experiment 5, the Phyto-low animals demonstrated greater immobility and lower serotonin levels. These differences were reversed following equol injection as time immobile significantly decreased and serum serotonin levels significantly increased in the Phyto-low animals. This study is the first to test and report equol's potential effectiveness as an antidepressant using the PFST. The testing of other isoflavones such as quercitin or resveratrol in the PFST, while few in number, demonstrate that isoflavones have potential as antidepressant treatments [36,37].

Equol's antidepressant potential demonstrated in the PFST, may be attributed to equol's estrogenic actions in the brain. ERβ is found in four brain regions associated with behavioral and mood disorders, the frontal cortex, amygdala, hippocampus, and the hypothalamic region [38-41]. Following dietary isoflavone exposure equol levels in the frontal cortex are highest followed by hypothalamus, amygdala, and the hippocampus [42], demonstrating an ability for equol to concentrate in these regions. Additionally, though the equol levels in the amygdala, hypothalamus, and hippocampus are lower, equol is still the primary isoflavone found in these regions, representing over 80 to 90% of the total isoflavone content of these brain areas [42].

Remarkably, equol could potentially affect the concentration of serotonin. For example, Dewar et al., reported that equol was an effective inhibitor of rat liver monoamine oxidase [43]. Since monoamine oxidase is responsible for the deamination of monoamines, including serotonin, this may account for the increase levels of this important neurotransmitter associated with the positive shift in depressive-like behaviors observed in the present study. Notably, this was shown in NOF females fed a soy-rich diet or equol administration alone in NOF females fed a low-soy diet. Specifically, the raphe nucles of the brain, which produces serotonin expresses ERβ [44]. Lower serotonin levels are associated with depression in humans [45-48]. Furthermore, the relationship between serum and CSF serotonin levels indicates potential for decreased serum serotonin concentrations to signify depression [45]. Equol's ability to bind ERβ's in various brain regions may have increased serotonin levels and improved mobility intervals in the PFST suggesting equol's potential as an antidepressant agent that represents quite novel results.

It is noteworthy that ERβ-deficient mice express increased anxiety [49] while administration of specific ERβ modulators in animal studies decrease anxiety and depression [40,41,50,51]. Furthermore, in human studies of depressed subjects, fluoxetine treatment increased serum serotonin levels by 1.7-fold [52], a value similar to that seen in experiment 5 in this study which may suggest a similar mechanism of action or potentially an increase in serotonin with enhanced catecholamine levels as reported by others in human studies [53]. In any event, further study for the potential of equol's antidepressant actions is warranted.

However, the use of animals with varying ovarian status demonstrated that equol appears to have a defined range of effectiveness. For instance, ovarian status plays a role in equol treatment effectiveness. Equol is ineffective at decreasing depressive-related behavior in intact females or females ovariectomized shortly after puberty. Experiments 1-4 all displayed no significant differences in depressive-related behavior as measured by the PFST. Only experiment 5 showed any significant differences. Conversely, from this study it appears that equol has potential as an anti-obesity treatment regardless of ovarian status; however, as an antidepressant it appears to be effective only following NOF.

Menopause/ovarian failure is a normal part of the aging process [54]. Depression and obesity are associated with menopause and affect both health and quality of life [3,55]. Central obesity increases risk for many menopausal conditions such as cardiovascular disease or breast cancer [56,57]. Depression can cause menopausal symptoms and associated conditions to feel and appear more serious [55]. Additionally, more women are entering menopause prematurely due to either bilateral oophorectomy or cancer treatment [2,55,58,59]. Severity of menopausal symptoms and the risk of certain diseases and conditions increase as age of menopause decreases [60]. Furthermore, the five experiments in this study indicate that timing and manner of ovarian failure can affect treatment response. However, the extrapolation from animals to humans is not known.

## Conclusions

The current study demonstrates that equol and/or isoflavonoids can decrease body weight, abdominal WAT, and depressive-related behavior dependent on age at initiation of diet and changes in ovarian status. However, equol's effectiveness as an antidepressant may be dependent upon the changes/timing of ovarian status with aging. This study demonstrated that equol's beneficial effects are most clearly seen in female rats that have undergone NOF. Further studies determining equol's exact mechanisms of action and potential as an antidepressant are required.

## Competing interests

The authors declare that they have no competing interests.

## Authors' contributions

CB- participated in the experiment design, performed the experiments, analyzed the data and authored the text, etc; KMF- participated in the experiment design, performed the experiments, analyzed the data; KDRS- performed the isoflavone blood analysis; TDL- performed the experiments and data analysis of the Porsolt results; EDL- participated in the experimental design, performed the experiments, data analysis, authoring of the text, etc. All authors read and approved the final manuscript.
